# Engineered Dwarf Male-Sterile Rice: A Promising Genetic Tool for Facilitating Recurrent Selection in Rice

**DOI:** 10.3389/fpls.2017.02132

**Published:** 2017-12-13

**Authors:** Afsana Ansari, Chunlian Wang, Jian Wang, Fujun Wang, Piqing Liu, Ying Gao, Yongchao Tang, Kaijun Zhao

**Affiliations:** ^1^National Key Facility for Crop Gene Resources and Genetic Improvement, Institute of Crop Science, Chinese Academy of Agriculture Sciences, Beijing, China; ^2^Crop Institute of Ningxia Academy of Agriculture and Forestry Sciences, Yingchuan, China; ^3^College of Agriculture, Guangxi University, Nanning, China

**Keywords:** rice breeding, dwarf, male-sterile, emasculation, recurrent selection, RNAi, multiple gene manipulation

## Abstract

Rice is a crop feeding half of the world’s population. With the continuous raise of yield potential via genetic improvement, rice breeding has entered an era where multiple genes conferring complex traits must be efficiently manipulated to increase rice yield further. Recurrent selection is a sound strategy for manipulating multiple genes and it has been successfully performed in allogamous crops. However, the difficulties in emasculation and hand pollination had obstructed efficient use of recurrent selection in autogamous rice. Here, we report development of the dwarf male-sterile rice that can facilitate recurrent selection in rice breeding. We adopted RNAi technology to synergistically regulate rice plant height and male fertility to create the dwarf male-sterile rice. The RNAi construct pTCK-EGGE, targeting the *OsGA20ox2* and *OsEAT1* genes, was constructed and used to transform rice via *Agrobacterium*-mediated transformation. The transgenic T0 plants showing largely reduced plant height and complete male-sterile phenotypes were designated as the dwarf male-sterile plants. Progenies of the dwarf male-sterile plants were obtained by pollinating them with pollens from the wild-type. In the T1 and T2 populations, half of the plants were still dwarf male-sterile; the other half displayed normal plant height and male fertility which were designated as tall and male-fertile plants. The tall and male-fertile plants are transgene-free and can be self-pollinated to generate new varieties. Since emasculation and hand pollination for dwarf male-sterile rice plants is no longer needed, the dwarf male-sterile rice can be used to perform recurrent selection in rice. A dwarf male-sterile rice-based recurrent selection model has been proposed.

## Introduction

Rice (*Oryza sativa* L.) is a staple crop that feed more than three billion people, nearly half of the world’s population (Elert, 2014). Since the world population is still growing (Gerland et al., 2014), and the arable land and water resources for rice production are decreasing due to urban expansion (Seto et al., 2012) and climate change (Wheeler and von Braun, 2013), there is therefore an urgent need for new breeding strategies to increase rice production in future (Ray et al., 2013).

In the history of rice breeding, there were two quantum leaps regarding enhancement of rice yield: from late 1950s to early 1970s, the conventional breeding technologies characterized with “Green Revolution” have made terrific contributions in doubling the world rice production (Khush et al., 2001); during 1970s–1980s, the hybrid rice breeding technologies pioneered by China enhanced the rice yield potential by some 25% (Peng et al., 2008). Thereafter, the so-called new plant type and super high-yielding rice (super rice) breeding programs have been conducted and significant progresses have been achieved in raising rice yield potential (Cheng et al., 2007; Peng et al., 2008). However, the actual rice yield seems to be approaching a ceiling in recent decade (Mann, 1999; Zhang et al., 2014).

Nowadays, it has been well recognized that many economically important traits of rice, such as yield, grain quality and tolerance to abiotic stresses, are controlled by a large number of loci; while those quantitative traits are of growing importance in future breeding as favorable alleles for most simply inherited traits are already prevalent and often fixed in elite germplasm (Varshney et al., 2012). Therefore, new breeding technologies that can efficiently manipulate multiple genes are urgently required.

Recurrent selection is one of the strategies for synergistically manipulating multiple genes in plant and animal breeding. RS refers to selection methods that are cyclical and conducted in a repetitive manner to gradually increase the frequency of favorable genes/alleles of quantitatively inherited traits in a population (Hallauer and Darrah, 1985). Historically, RS is an old concept; the detailed description of RS was provided by Jenkins (1940). Over the past several decades, RS has been successfully carried out in breeding of maize and other allogamous crops where controlled intermating and seed production is simpler (Hallauer and Darrah, 1985; Hallauer et al., 2010). However, the need of emasculation and hand pollination had obstructed the efficient use of RS in breeding of autogamous species. Recently, this situation is changing due to the development of the DMS wheat which links the dominant dwarf gene *Rht-D1c* and male-sterile gene *Ms2* (Liu and Yang, 1991). The DMS wheat-based RS breeding platform has been widely adopted in wheat breeding programs in China, which have produced more than 40 new varieties planted on larger than 12 mha (Ni et al., 2017; Xia et al., 2017). However, the problem of emasculation and hand pollination in rice RS breeding remains to be solved.

RNA interference (RNAi) is a post-transcriptional gene-silencing phenomenon induced by double-stranded RNA (dsRNA). When a designer hairpin RNAi construct is introduced into a plant cell, the expressed hairpin dsRNAs are cleaved into short (∼20–25 nucleotides) dsRNA fragments called small interfering RNA (siRNA) by the endoribonuclease Dicer. Each siRNA is subsequently unwound into single-stranded RNAs, the passenger (sense) strand, and the guide (antisense) strand. The passenger strand is degraded and the guide strand is incorporated into the RNA-induced silencing complex (RISC). After integration into the RISC, the guide strands base-pair to portions of their complementary mRNA and allow the RISC cleaves the mRNA, thereby preventing it from being used as a translation template, and finally the target gene is silenced (Hannon, 2002; Jinek and Doudna, 2009). RNAi technology has been approved a powerful tool for suppressing expression of a target gene specifically and is of practical usability in genetic improvement of crops (Kusaba, 2005; Ali et al., 2010, 2013a,b; Saurabh et al., 2014; Xu et al., 2016).

Rice plant height is associated with endogenous biologically active gibberellins (GAs) (Ashikari et al., 2003). *OsGA20ox2* encodes a regulatory enzyme for the syntheses of biologically active GAs in rice and mutation in *OsGA20ox2* causes the semi-dwarf phenotype (Spielmeyer et al., 2002). We have previously adopted RNAi technology to regulate the plant height by suppressing expression of the *OsGA20ox2* gene in rice, where a designer hairpin RNAi vector pCQK2 targeting the *OsGA20ox2* gene was construct and introduced into rice, resulting in the RNAi-dwarfed plants with reduced plant height, but comparable grain yield to that of the wild-type (Qiao et al., 2007).

Pollen fertility is crucial for rice seed production. The formation of microspores and their development into mature pollen grains require cooperative interactions between gametophytic (microspores) and sporophytic (anther wall) cells, in which the tapetum plays the most crucial role (Ma, 2005). Tapetal cells comprise the innermost anther wall layer, which encloses male reproductive cells, and undergo programmed cell death (PCD)-triggered degradation after the meiosis of microspore mother cells. PCD and the consequent cellular degradation of the tapetum must occur during the proper anther developmental stages for microspore development and pollen wall maturation. Premature, or delayed tapetal PCD and cellular degeneration cause pollen sterility (Li et al., 2006). Several rice genes including *OsEAT1* are known to be involved in the differentiation, degeneration, and function of the tapetum and microspore development (Guo and Liu, 2012; Niu et al., 2013). *OsEAT1* encodes a basic helix-loop-helix transcription factor that positively regulates PCD in tapetal cells during rice male reproductive development; mutation of *OsEAT1* cause delayed tapetal cell death and aborted pollen formation, resulting in complete male sterility (Niu et al., 2013).

On the other hand, male-sterile germplasms have been constantly exploited to improve the rice productivity by hybrid rice breeding (Cheng et al., 2007). Recently, a male sterility system has been engineered for hybrid rice breeding and seed production (Chang et al., 2016). Here, we report development of DMS rice by adopting RNAi technology to synergistically suppress expression of the *OsGA20ox2* and *OsEAT1* genes, resulting in co-inherited semi-dwarf and male-sterile phenotypes in rice plants. This DMS rice can be used as a genetic tool for RS breeding in rice.

## Materials and Methods

### Plant Materials and Bacterial Strains

The *Japonica* rice varieties NJ36, JJ88, Z0201 and their transgenic plants were used in this study. Rice seeds were immersed in water for 2 days, sown in seed beds and grown for 1 month and then transplanted to the field with normal daylight illumination in Beijing or Sanya, China. *Escherichia coli* DH5a, DH10B and *Agrobacterium tumefaciens* strain EHA105 were used for cloning and transformation experiments, respectively.

### Primers

Nucleotides for all primers used for PCR and qRT-PCR analyses are provided in Supplementary Table S3.

### Construction of Hairpin RNAi Vector pTCK-RGGR

The GA20 fragment in antisense orientation of *OsGA20ox2* (GenBank:AF465255.1) was PCR-amplified from rice variety QX1 (Qiao et al., 2007) by using primer pairs GaF-Rts and GaR-Sp (Supplementary Table S3), A *Spe*I restriction site at one end of the fragment has been introduced via the primer GaR-Sp. The RTS fragment in antisense orientation of *OsRTS* (GenBank: U12171.1) was PCR-amplified from rice variety NJ36 by using primers RtsF-Sa and RtsR-Ga (Supplementary Table S3). A *Sac*I restriction site at one end of the fragment has been introduced via the primer RtsF-Sa. The GA20 and RTS fragments in antisense orientation were joined by overlap PCR with primers RtsF-Sa and GaR-Sp (Supplementary Table S3). The joined RTS-GA20 fragment in sense orientation was PCR-amplified from the joined GA20-RTS fragment using primers RtsF-B/GaR-K, and the *BamH*I and *Kpn*I restriction sites at the ends of the fragment have been introduced. The joined GA20-RTS fragment in antisense orientation was cloned into the backbone vector pTCK303 (Wang et al., 2004) at the *Spe*I and *Sac*I restriction sites, resulted in the intermediate vector pTCK-GR. Finally, the joined RTS-GA20 fragment in sense orientation was cloned into the intermediate vector pTCK-GR through the *BamH*I and *Kpn*I restriction sites, resulted in the hairpin RNAi vector pTCK-RGGR.

### Construction of Hairpin RNAi Vector pTCK-EGGE

The target regions of *OsGA20ox2* (GenBank: AF465255) and *OsEAT1* (LOC_Os04g51070^1^) were PCR-amplified and jointed via overlap PCR to generate the antisense fragment EGR and the sense fragment EGF with appropriate restriction sites introduced at the ends (**Figure 3** and Supplementary Table S4). The fragments EGF and EGR were cloned into the backbone vector pTCK303 (Wang et al., 2004) at the corresponding restriction sites, resulting in the RNAi construct pTCK-EGGE used for genetic transformation of rice (**Figure 3**).

### Transformation of Rice

*Agrobacterium tumefaciens* strain EHA105 harboring the RNAi construct pTCK-RGGR or pTCK-EGGE was used for transformation of embryogenic calli generated from rice varieties NJ36, JJ88, and Z0201. The details of the transformation procedures were as described by Lin and Zhang (2005). Transgenic plants were selected on the medium containing 50 mg/L hygromycin. Hygromycin-resistant T0 plants were transplanted into soil and grown in greenhouse at approximately 28–35°C under natural sunlight.

### PCR Analysis of Putative Transgenic Rice Plants

The molecular characterization of putative transgenic plants involved PCR analyses. Genomic DNA was isolated from a small piece of the leaves collected from putative transgenic plants by using the SDS method (Stephen et al., 1983). The PCR reaction (20 μl) contained 100 ng of template DNA, 1 × KOD buffer, 0.6 mmol/L dNTPs, 0.15 mmol/L of each primer, and 0.75 unit of KOD FX DNA polymerase (TOYOBO), respectively. The PCR reaction was set up with an initial denaturation at 94°C for 3 min, followed by 35 cycles with denaturation at 94°C for 30 s, annealing at 60°C for 30 s, elongation at 72°C for 40 s and a final extension step at 72°C for 7 min. The paired primers (Supplementary Table S3) named as AJCF and AJCR were used to amplify expected 300 bp fragment from pTCK-EGGE (Supplementary Figure S1).

### qRT-PCR

RNA from different tissues of transgenic rice plants and the wild-type was isolated by using TRIZOL reagent (Invitrogen), and mRNA was purified with a messenger RNA kit (Qiagen). The RNA concentration and quality were measured using an ND-2000 Nanodrop spectrophotometer (Nanodrop Technologies, Thermo Scientific, Waltham, MA, United States). For qRT-PCR, 1.5 μg of RNA was reversely transcribed into cDNA using a FastQuant RT Kit (Tiangen Biotech, China) with random primers. cDNA derived from 37.5 ng of total RNA was used for each real-time PCR with gene-specific paired primers (EatQF1/EatQR1, Ga20QF/Ga20QR, Ga3QF/Ga3QR, and Cps1F/Cps1R) for *OsEAT1*, *OsGA20ox2*, *OsGA3ox2*, and *OsCPS1* genes, respectively (Supplementary Table S3) on a 7500 Real-Time PCR system using the SYBR SELECT MASTER MIX (Applied Biosystems). The rice ubiquitin gene *Ubi* was used as an internal control (primers UbqF and UbqR, Supplementary Table S3). The average threshold cycle (Ct) was used to determine the fold change of gene expression. The 2^-ΔΔC_T_^ method was used for relative quantification (Lievak and Schmittgen, 2001).

## Results

### DMS Rice Generated by Targeting *OsGA20ox2* and *OsRTS*

**FIGURE 1:**
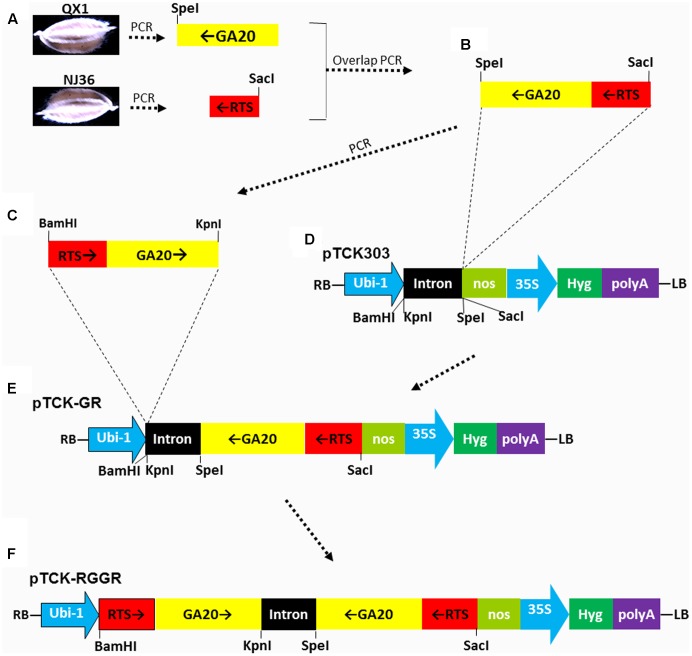
Schematic diagram of RNA interference (RNAi) vector pTCK-RGGR construction. Dash-dot arrows/lines indicate the relationship between the fragments or figure parts. Black arrows indicate the sense or antisense orientations of the DNA fragments RTS or GA20. **(A)** The GA20 fragment in antisense orientation of *OsGA20ox2* was PCR-amplified from rice variety QX1 by using primer pairs GaF-Rts and GaR-Sp (Supplementary Table S3). A *Spe*I restriction site at one end of the fragment has been introduced via the primer GaR-Sp. The RTS fragment in antisense orientation of *OsRTS* was PCR-amplified from rice variety NJ36 by using primers RtsF-Sa and RtsR-Ga (Supplementary Table S3). A *Sac*I restriction site at one end of the fragment has been introduced via the primer RtsF-Sa. **(B)** The GA20 and RTS fragments in antisense orientation were joined by overlap PCR with primers RtsF-Sa and GaR-Sp (Supplementary Table S3). **(C)** The joined RTS-GA20 fragment in sense orientation was PCR-amplified from the joined GA20-RTS fragment using primers RtsF-B/GaR-K, and the *BamH*I and *Kpn*I restriction sites at the ends of the fragment have been introduced via the primers. **(D)** The backbone vector pTCK303, with *BamH*I, *Kpn*I, *Spe*I, and *Sac*I restriction sites for exogenous fragment insertion. RB, right border; LB, left border; Ubi-1, the maize (*Zea mays*) ubiquitin promoter; nos, nos terminator; 35S, CAMV 35S promoter; Hyg, hygromycin phosphotransferase gene. The intron was a 478-bp rice intron amplified from rice (*Oryza* *sativa* L. cv. Zhonghua 10) (Wang et al., 2004). **(E)** Intermediate vector pTCK-GR generated by cloning the joined GA20-RTS fragment in antisense orientation into the backbone vector pTCK303 at the *Spe*I and *Sac*I restriction sites. **(F)** The joined RTS-GA20 fragment in sense orientation was cloned into the intermediate vector pTCK-GR through the *BamH*I and *Kpn*I restriction sites, resulted in the hairpin RNAi vector pTCK-RGGR.

We first constructed a RNAi construct designated as pTCK-RGGR (**Figure 1**) to simultaneously target the genes *OsGA20ox2* (GenBank:AF465255) and *OsRTS* (GenBank: U12171.1). *OsGA20ox2* encodes GA-20 oxidase-2, a regulatory enzyme for the syntheses of biologically active GAs in rice; the loss-of-function mutation in *OsGA20ox2* generates the well-known Green Revolution gene *sd-1*, which causes the semi-dwarfism phenotype of rice (Spielmeyer et al., 2002). *OsRTS* is a rice tapetum-specific gene required for male fertility; suppression of *OsRTS* can cause male-sterility in rice (Luo et al., 2006).

**FIGURE 2:**
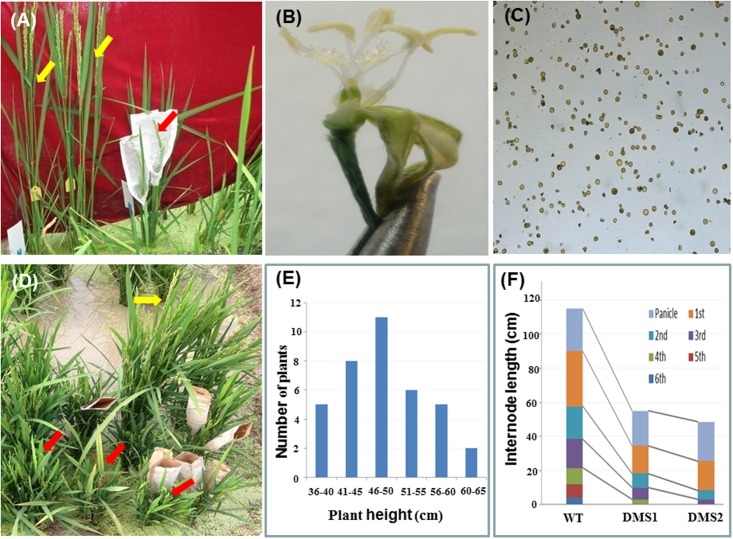
Phenotypes of pTCK-RGGR-transgenic rice plants. Red arrows indicate the positive pTCK-RGGR-transgenic rice plants which are dwarf and male-sterile (DMS). Yellow arrows indicate the non-transgenic rice plants (wild-type, WT) which are male-fertile and with normal plant height. **(A)** Phenotype comparison between the DMS plant (red arrow) and the WT (NJ36, yellow arrows) grown in field. **(B)** A spikelet from the DMS plant in **(A)** showing the small and pale anthers. **(C)** I_2_-IK stained pollens from the DMS plant in **(A)**, indicating that the poorly developed pollens are sterile. **(D)** Phenotype comparison between the DMS plants (red arrows) and the wild-type (Z0201, yellow arrow) grown in field. **(E)** Distribution of the 37 DMS plants generated in pTCK-RGGR-transformation of rice variety Z0201 in terms of plant height at ripening stage. **(F)** Comparison of length of plant height, panicle and internodes between the WT Z0201 and two DMS rice plants from **(E)**, showing that the dwarfism of the DMS plants are mainly due to the reduction of the lower internodes.

The RNAi construct pTCK-RGGR was used to transform embryogenic calli generated from the *Japonica* rice varieties NJ36 and JJ88 by *Agrobacterium*-mediated rice transformation; 11 and 39 positive transgenic rice plants were obtained, respectively. As expected, the pTCK-RGGR-transgenic T0 plants are dwarf and male-sterile, and therefore were designated as DMS rice plants (**Figure 2**). However, these DMS rice plants failed to set seeds after natural or artificial pollination with pollens from wild-type plants. This observation was confirmed by generating 37 positive transgenic rice plants by transforming another variety Zhongzuo0201 (Z0201) with the RNAi construct pTCK-RGGR (**Figure 2** and Supplementary Table S1).

### DMS Rice Generated by Targeting *OsGA20ox2* and *OsEAT1*

**FIGURE 3:**
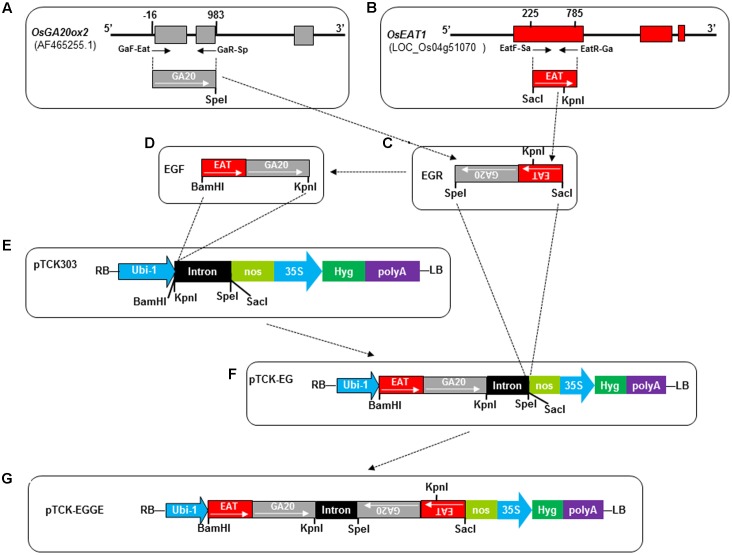
Construction of hairpin RNAi vector pTCK-EGGE. Dash-dot lines/arrows indicate the relationship between the fragments or figure parts. **(A)** Schematic diagram of *OsGA20ox2* gene structure and the fragment (GA20, 999 bp) for RNAi vector construction. The three exons are indicated by gray rectangles. The small arrows under the exons represent primers GaF-Eat and GaR-Sp whose 5′ positions related to the first nucleotide of the open reading frame are indicated by numbers above the exons. The restriction enzyme cutting site *Spe*I was introduced at the end of GA20 fragment. **(B)** Schematic diagram of *OsEAT1* gene structure and the fragment (EAT, 561 bp) for RNAi vector construction. The three exons are indicated by red rectangles. Numbers above the 1st exon indicate the 5′ positions of the primers EatF-Sa and EatR-Ga presented by small arrows. The *Sac*I site was introduced at the end of EAT by primer EatF-Sa. There is a native *BamH*I and *Kpn*I restriction site at 42th and 365th nucleotide of the 561 bp-EAT fragment, respectively. **(C)** The joint antisense fragment EGR generated by overlap PCR with primers GaR-Sp and EatF-Sa. **(D)** The joint sense fragment EGF generated from the antisense fragment EGR by overlap PCR. The internal *Kpn*I restriction site GGTACC in EGR was mutated as GGTCAG (Supplementary Table S4). **(E)** Intermediate vector pTCK303, with *BamH*I, *Kpn*I, *Spe*I, and *Sac*I restriction sites for exogenous fragment insertion. RB, right border; LB, left border; Ubi-1, the maize (*Zea mays*) ubiquitin promoter; nos, nos terminator; 35S, CAMV 35S promoter; Hyg, hygromycin phosphotransferase gene. The intron was a 478-bp rice intron amplified from rice (Wang et al., 2004). **(F)** Intermediate vector pTCK-EG generated by cloning the sense fragment EGF into the backbone vector pTCK303 at the *BamH*I and *Kpn*I restriction sites. **(G)** Structure of the hairpin RNAi vector pTCK-EGGE.

Since the pTCK-RGGR-transgenic T0 plants failed to set seeds after artificial pollination, we speculated that the RNAi suppression of *OsRTS* expression would affect the female-fertility of the DMS rice plants generated by the pTCK-RGGR construct (**Figure 2**). Therefore, we used the *OsEAT1 (ETERNAL TAPETUM 1)* gene (LOC_Os04g51070) to replace *OsRTS* and constructed another RNAi construct designated as pTCK-EGGE (**Figure 3**). EAT1 is a major regulator in rice microspore development; mutation of *OsEAT1* can cause male-sterility in rice (Niu et al., 2013).

**FIGURE 4:**
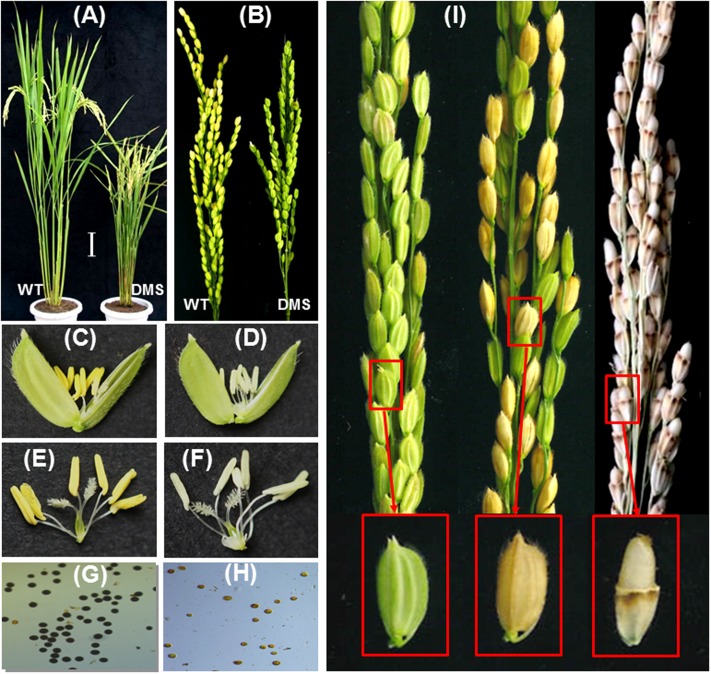
Phenotypic characterization of pTCK-EGGE-induced DMS rice. **(A)** Plant phenotype of WT and DMS rice plants. Bar = 10 cm. **(B)** Panicles of WT and DMS rice plants. **(C,D)** Spikelets of the WT and DMS rice plants, respectively. **(E,F)** Anther/stigma of the wild-type and DMS rice plants, respectively. **(G,H)** Pollens of the WT and DMS rice plants, respectively. **(I)** Parts of DMS rice panicles showing complete sterile spikelets (left, without pollination), 30–50% fertile spikelets (middle, with natural pollination) and fertile spikelets (right, with artificial pollination). Enlarged spikelets were shown.

The RNAi construct pTCK-EGGE was then used to transform the *Japonica* rice variety Z0201 by *Agrobacterium*-mediated rice transformation. The regenerated plants were analyzed by PCR with gene-specific primers (Supplementary Figure S1A) and 46 positive transgenic rice plants were obtained. The plant height of the pTCK-EGGE-transgenic DMS plants in T0 generation ranged in 50–80 cm (Supplementary Figure S2), while the plant height of wild-type plants is about 113 cm (Supplementary Table S2). A representative DMS plant was shown in **Figure 4A**. The panicles of the DMS plants were somewhat shorter than the wild-type, but the grain morphology was similar to the wild-type (**Figure 4B**). Most of the DMS plants had more tillers and a shorter heading stage than the wild-type plants (Supplementary Table S2). Compared with the wild-type, the anthers of the DMS plants were smaller and paler (**Figures 4C–F**), with no pollen or only a few sterile pollens (**Figures 4G,H**). This made the DMS plants completely sterile in condition without exogenous pollens (**Figure 4I**, left). Fortunately, the seed-setting rate of the pTCK-EGGE-transgenic DMS rice was usually 30–50% in condition of open-pollination (**Figure 4I**, middle) and could be higher than 90% upon hand-pollination (**Figure 4I**, right), indicating the pTCK-EGGE-induced RNAi specifically suppresses the pollen fertility, but no interruption to the stigma fertility of the DMS plants.

### Inheritance of the DMS Traits

Selected pTCK-EGGE-transgenic DMS rice plants in T0 or T1 generations were cross-pollinated with pollens from the wild-type plants, resulting in T1 or T2 lines. Existence of the RNAi construct in DMS rice plants from T1 and T2 populations have been confirmed by PCR analysis (Supplementary Figure S1). DMS rice plants with a plant height of 50–60 cm were preferentially selected for cross-pollination, resulting in more DMS plants with that plant height range in T2 populations (Supplementary Figure S2). In most of the T1 populations, about half of the plants were DMS; the other half displayed the normal plant height and male fertility as the wild-type and therefore they were designated as TMF plants. The DMS/TMF plants fitted 1:1 ratio statistically (**Table 1**). Such phenotypic segregation pattern has also been observed in the T2 generation (**Table 1**), indicating the hairpin RNAi structure consisting of the jointed fragments from *OsGA20ox2* and *OsEAT1* can be stably inherited as a single locus. In addition, other traits such as panicle length, effective tillers per plant, spikelets per panicle and days to heading of the DMS rice plants were faithfully inherited to the following generations (Supplementary Table S2).

**Table 1:** Segregation pattern of offspring from DMS rice plants.

T0 generation plants					T1 generation (number of plants)				T2 generation (number of plants)	χ^2^
		Total	DMS	TMF	DMS:TMF	Total	DMS	TMF	DMS:TMF	1:1
TD21	1	1	1	0	1:0.0	8	3	5	1:0.6	^∗^
TD39	1	26	13	13	1:1.0	159	76	83	1:0.9	^∗^
TD46	1	63	32	31	1:1.0	112	55	57	1:1.0	^∗^
TD140	1	3	1	2	1:0.5	17	7	10	1:0.7	^∗^
Total	4	93	47	46	1:1.0	296	141	155	1:0.9	^∗^

*DMS, dwarf and male-sterile plants; TMF, tall and male-fertile plants. ^∗^ 5% level of significance*.

### Relationship between the DMS Traits and RNAi Suppression

**FIGURE 5:**
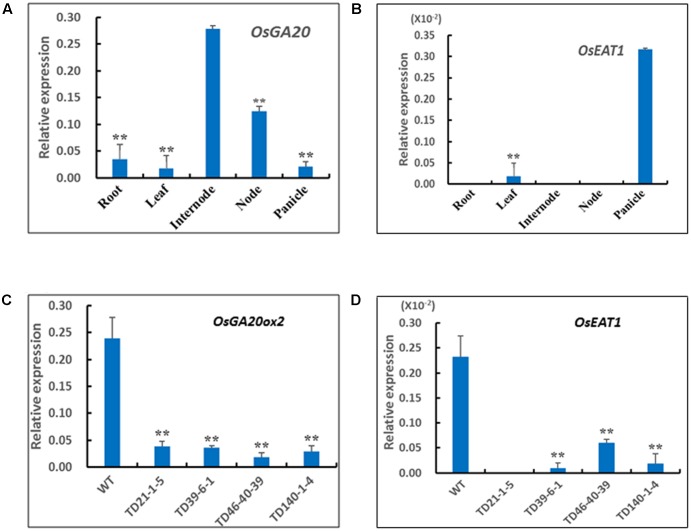
Expression patterns of *OsGA20ox2* and *OsEAT1* in DMS rice and the WT Z0201. **(A)** Tissue-specific expression pattern of *OsGA20ox2* in Z0201. **(B)** Tissue-specific expression pattern of *OsEAT1* in Z0201. **(C)** Relative expression levels of *OsGA20ox2* in stem internodes of transgenic DMS rice lines and WT at heading stage. **(D)** Relative expression levels of *OsEAT1* in panicles of transgenic DMS rice lines and WT at heading stage. The rice *Ubi* gene was used as an internal control. The data are shown as mean values ± SD from three replicates. The asterisks indicate significant difference (one-tail *t*-test, compared with WT, ^∗∗^*P* < 0.01; ^∗^*P* < 0.05).

To examine the relationship between the RNAi suppression and the phenotypes of DMS rice, we performed quantitative real-time PCR to analyze expression levels of endogenous *OsGA20ox2* and *OsEAT1* genes in the plants. qPCR analysis with the gene-specific primers Ga20QF and Ga20QR (Supplementary Table S3) revealed that the *OsGA20ox2* gene was highly transcribed in stem internodes of the wild-type Z0201 (**Figure 5A**), thus we analyzed expression levels of the *OsGA20ox2* in stem internodes of randomly selected DMS rice plants. Results showed that the *OsGA20ox2* gene expression in the DMS rice plants has been drastically suppressed (**Figure 5C**). In addition, we also checked the transcript levels of *OsGA3ox2* and *OsCPS1*, another two regulatory genes for GA biosynthesis (Itoh et al., 2001; Toyomasu et al., 2015). qPCR results showed that expression level of *OsGA3ox2* or *OsCPS1* in the RNAi transgenic lines is similar to that of the wild-type Z0201 (Supplementary Figure S3), indicating that the RNAi construct pTCK-EGGE specifically suppressed the expression of *OsGA20ox2*; this is in coincidence with observations in our previous study (Qiao et al., 2007).

Since the *OsEAT1* gene was almost exclusively expressed in panicles of the wild-type (**Figure 5B**), we examined *OsEAT1* expression levels in panicles of the selected DMS plants, and found that the transcripts of *OsEAT1* gene have also been drastically reduced in the DMS rice plants (**Figure 5D**). These results are in coincidence with the phenotypes of reduced plant height and male-sterility of the DMS plants (**Table 2**).

**Table 2:** Morphological characters of DMS rice plants used for gene expression analysis.

Plant	PH	MS	ET/P	PL	SP/Pn	DTH
TD21-1-5	65	No/undeveloped pollen (100%S)	7	25.4	210	112
TD39-6-1	56	No/undeveloped pollen (100%S)	7	21.3	180	90
TD46-40-39	52	No/undeveloped pollen (100%S)	9	21.7	190	79
TD140-1-4	52	No/undeveloped pollen (100%S)	7	20.3	197	100
Wild-type	114	Fertile	5	24.1	212	112

*PH, plant height (cm); MS, male-sterility; ET/P, number of effective tiller per plant; PL, panicle length (cm); SP/Pn, number of spikelet per panicle; DTH, days to heading*.

## Discussion

In breeding of allogamous crops like maize, RS has been approved an efficient approach for manipulating multiple genes synergistically to increase the frequency of favorable genes/alleles of quantitatively inherited traits, but RS has not been widely used in the autogamous crop rice because of the difficulties in emasculation and hand pollination. The development of DMS wheat by genetically linking the dominant dwarf gene *Rht-D1c* and male-sterile gene *Ms2* had provided an efficient RS-breeding platform from which more than 40 new varieties have been bred and cultivated in China (Ni et al., 2017; Xia et al., 2017). In this study, we developed the DMS rice by using RNAi technology to synergistically regulate rice plant height and male fertility. The RNAi construct pTCK-EGGE can synergistically suppress the transcription of both the targeted genes *OsGA20ox2* and *OsEAT1*, resulting in the semi-dwarf and male-sterile phenotypes in a single rice plant. We have analyzed another two GA-biosynthesis regulatory genes (*OsGA3ox2* and *OsCPS1*) by qPCR and found that the expression levels of *OsGA3ox2* and *OsCPS1* in the DMS rice lines are similar with those of the wild-type plants (Supplementary Figure S3), indicating a specific suppression of pTCK-EGGE on the expression of *OsGA20ox2*, which is in coincidence with observations in our previous study (Qiao et al., 2007).

Like the DMS wheat breeding system, emasculation and hand pollination for DMS rice plants is no longer needed; crossing between DMS rice plants and other rice varieties can be easily done in breeding, and this will definitely save labor and increase working efficiency. In this context, DMS rice can provide a favorable tool for efficient pyramiding of multiple genes in rice breeding.

**FIGURE 6:**
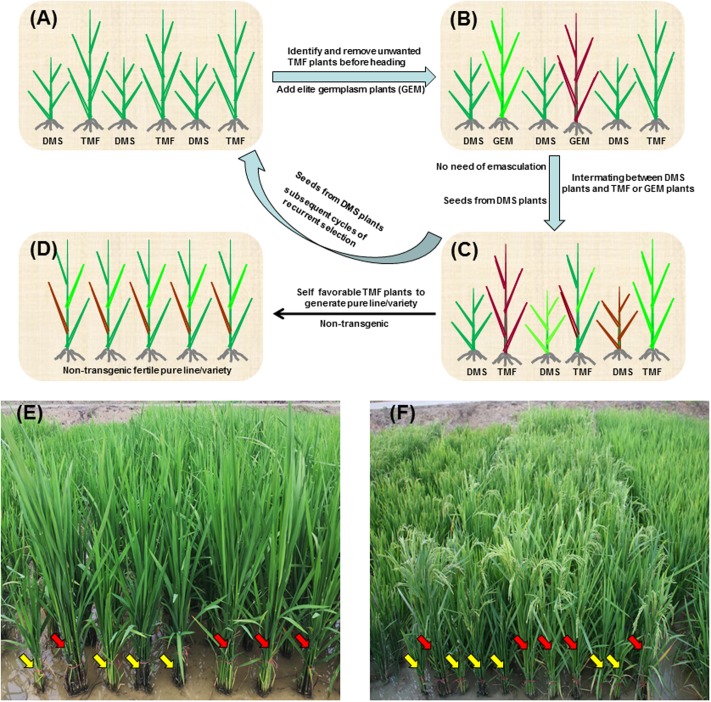
Schematic representation of DMS rice-based RS breeding. **(A)** Initial DMS rice population represented by six plants. Half of the plants are DMS plants and the other half are TMF plants. The DMS plants are distinguishable from TMF plants by plant height before heading stage. **(B)** Intermating population for RS. Unfavorable or unwanted plants in the initial DMS rice population are removed and germplasm (GER) plants of interest are introduced before heading. At flowering stage, the TMF and GER plants will randomly pollinate the DMS plants. Since DMS plants are completely male-sterile, emasculation is exempted. **(C)** Advanced DMS rice population generated from seeds of the DMS plants in the intermating population. Advanced DMS rice population can serve as the starting population of subsequent cycles of RS. **(D)** New variety population. After some cycles of RS, multiple genes/traits could be pyramided in some advanced TMF plants. Favorable TMF plants can be self-pollinated to generate non-transgenic pure lines/varieties. **(E,F)** A real initial DMS rice population at tillering stage **(E)** and flowering stage **(F)**. Yellow and red arrows indicate the DMS and TMF plants, respectively.

More importantly, the DMS rice population can be easily used for RS breeding of rice (**Figure 6**). Since the dwarfism phenotype of DMS rice enables early discriminating between the DMS and TMF plants in the DMS rice population (**Figures 6A,E**), unwanted TMF plants as well as undesirable DMS plants can be removed before heading, while other germplasm plants of interest can be introduced into the population. At flowering stage, the TMF and introduced germplasm plants can randomly pollinate the DMS plants where emasculation is exempted since the DMS plants are completely male-sterile (**Figure 6F**). Seeds harvested from the DMS plants will generate progeny population in which again half of the plants are DMS plants and the other half are TMF plants. Such random crossing between the DMS plants and the TMF or introduced new germplasm plants can be repeated for infinite cycles. In this way, efficient RS breeding of autogamous crop rice can be realized via the DMS rice populations (**Figures 6A–C**). The RS procedures will enhance the efficiency of crossing between different genotypes and facilitate pyramiding of multiple genes in the populations. After some cycles of RS, multiple favorable genes/traits will be pyramided in TMF plants in the advanced DMS rice population, and those advanced favorable TMF plants can be self-pollinated to generate pure lines that can be tested for varieties (**Figure 6D**). Notably, due to the inheritance nature or segregation pattern of the RNAi construct in the DMS rice population, the pure lines or varieties derived from the TMF plants are transgene-free. Therefore, there should be no biosafety concerns for the varieties released from the DMS rice-based RS breeding.

## Author Contributions

KZ conceived and designed the research. JW and FW designed some of the experiments. JW, CW, PL, AA, and YG performed experiments on pTCK-RGGR and its transgenic plants. AA, CW, FW, and YT performed experiments on pTCK-EGGE and its transgenic plants. KZ and AA wrote the manuscript.

## Conflict of Interest Statement

Institute of Crop Science, CAAS and Ningxia Academy of Agriculture and Forestry Sciences have filed a patent application (201110045310.1) on behalf of KZ, JW, CW, YG, and PL based on the strategy and results on pTCK-RGGR. The other authors declare that the research was conducted in the absence of any commercial or financial relationships that could be construed as a potential conflict of interest.

## Footnotes

1 http://rice.plantbiology.msu.edu/cgi-bin/ORF_infopage.cgi

## Abbreviations

DMS: dwarf male-sterile
RS: recurrent selection
TMF: tall and male-fertile
