# Supplementary material for: Engineered Dwarf Male-Sterile Rice: A Promising Genetic Tool for Facilitating Recurrent Selection in Rice
Source: Front Plant Sci. 2017 Dec 13;8:2132. doi: 10.3389/fpls.2017.02132 (PMC5733493; doi:10.3389/fpls.2017.02132)
Supplement: Supplementary file 1 [file Data_Sheet_1.pdf]

Supplementary Materials for

Engineered Dwarf Male-Sterile Rice: A Promising Genetic Tool for Facilitating Recurrent Selection in Rice

Afsana Ansari[1,4,†], Chunlian Wang[1,†], Jian Wang[1,2,†], Fujun Wang[1,3,†], Piqing Liu[3], Ying Gao[1], Yongchao Tang[1] and Kaijun Zhao[1*]

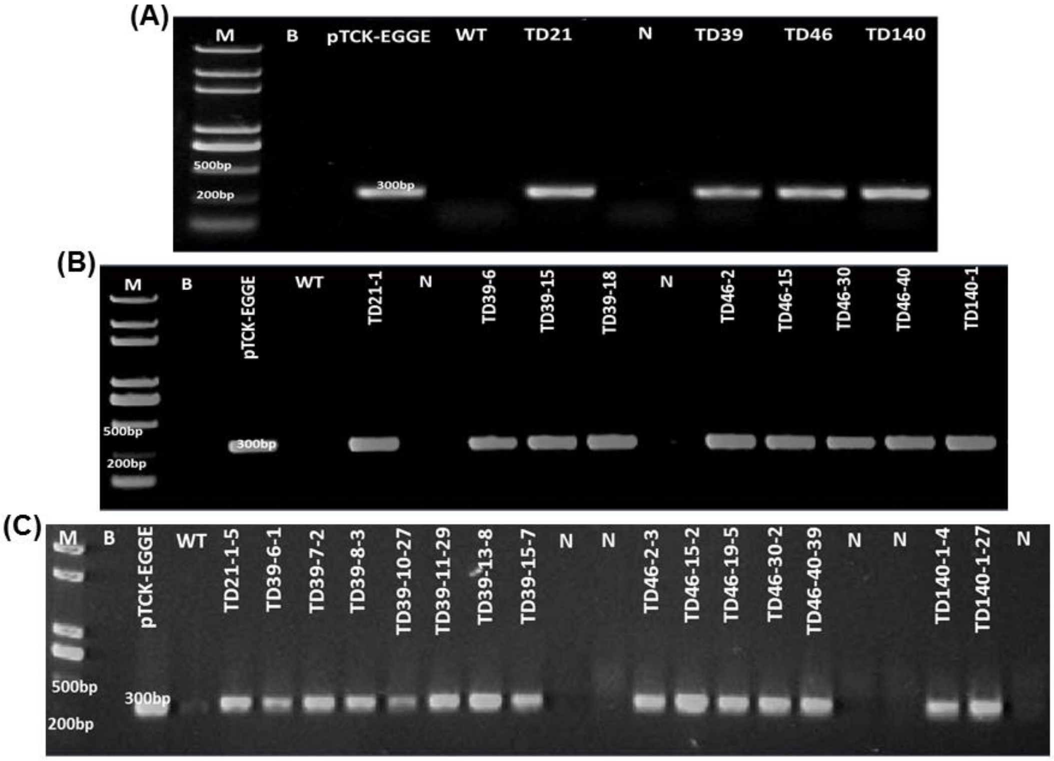

Supplementary Figure S1 PCR analysis of transgenic rice plants with gene-specific primers AJCF/AJCR. **(A-C)** T0, T1 and T2 generations, respectively. Lanes: M, 2000 plus DNA ladder, B, blank, WT, non-transformed control plant, pTCK-EGGE, positive control (plasmid), N, negative plants.

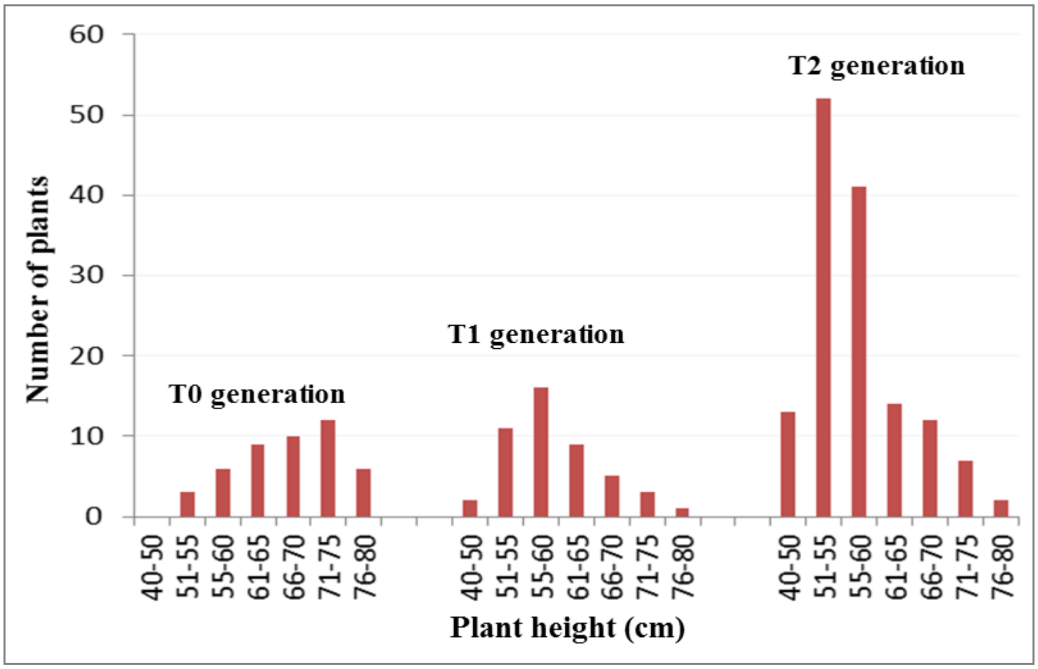

Supplementary Figure S2 Distribution of pTCK-EGGE induced DMS rice plants in terms of plant height at ripening stage in T0, T1 and T2 generations.

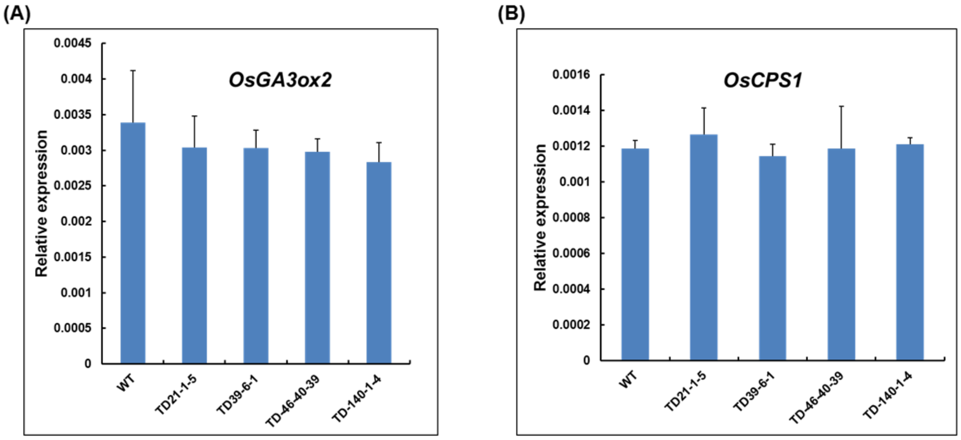

Supplementary Figure S3 Expression patterns of *OsGA3ox2* and *OsCPS1* in DMS rice and the wild-type Z0201. **(A)** Relative expression levels of *OsGA3ox2* in stem internodes of transgenic DMS rice lines and wild type (WT) at heading stage. **(B)** Relative expression levels of *OsCPS1* in stem internodes of transgenic DMS rice lines and wild type (WT) at heading stage. The rice *Ubi* gene was used as an internal control. The data are shown as mean values ± SD from three replicates. No significant difference is detected (one-tail t-test, compared with WT).

Supplementary Table S1 Comparison of some morphological characters between pTCK-RGGR-transgenic DMS rice and wild-type plants

Traits	Wild type	pTCK-RGGR-transgenic Plants
PH	114.67± 2.52	48.67±3.6**
MS	Fertile	No/undeveloped pollen**
ET/P	8.67±0.58	15.33±2.52*
PL	25.83±1.04	22.17±1.04*
SP/Pn	210.00±10.00	171.67±10.41*

Mean values calculated from 3 plants of wild type (Z0201) or pTCK-RGGR-transgenic Plants (T0) as per the standard evaluation system of rice at IRRI; Significance was determined by t-test; Values were presented as mean ± SE; Length is in cm; PH, Plant height (cm); MS, Male sterility; ET/P, Number of effective tillers per plant; PL, Panicle length (cm); SP/Pn, Number of spikelets per panicle. *, 5% level of significance; **, 1% level of significance.

Supplementary Table S2 Morphological characters of pTCK-EGGE-induced DMS rice plants

	T0 generation (in Beijing)			T1 generation (in Sanya)			T2 generation (in Beijing)		
	WT	DMS Plants		WT	DMS Plants		WT	DMS Plants	
PH	112.67±11.59	69.9±10.7	**	115.67±10.06	71.33±9.71	**	111.00±7.87	66.58±12.86	**
MS	Fertile	No/undeveloped pollen	**	Fertile	No/undeveloped /sterile pollen	**	Fertile	No/undeveloped /sterile pollen	**
ET/P	9.67±2.08	11.2±6.34	NS	4.66±0.57	6.21±3.85	NS	4.25±1.26	6.04±2.86	NS
PL	25.83±1.04	24.50±2.72	NS	25.66±2.08	23.41±3.85	NS	23.25±3.28	20.4±4.11	NS
SP/Pn	210.00±10.00	186.30±20.28	*	198.33±10.41	183.75±37.96	NS	185.5±17.82	166.38±43.86	NS
DTH	NA	NA		79.00±2.00	72.16±3.47	**	109.00±9.59	98.83±15.44	NS
DTM	NA	NA		105.00±2.00	98.00±3.43	**	134.75±9.91	124.89±15.46	NS

Mean values calculated from 3 plants of wild type (WT), all DMS plants of pTCK-EGGE RNAi lines from T0, T1 and T2 generation as per the standard evaluation system of rice at IRRI; Statistical significant was determined by t-test; Values were presented as mean ± SE; Length is in cm; NA, not available. PH, Plant height (cm); MS, Male sterililty; ET/P, Number of effective tiller per plant; PL, Panicle length (cm); SP/Pn, Number of spikelet per panicle; DTH, Days to heading; DTM, Days to maturity; *, 5% level of significance; **, 1% level of significance; NS, Non-significant.

Supplementary Table S3 Primers used in this study

Name	Sequence (from 5' to 3')	Feature and for experiment
GaF-Rts	TGCTCCACCTCGCTCTGATTTCTCATCTCCAATCTCATGG	*OsRTS*-adapter added, for fragment GA20
GaR-Sp	CCACTAGTACCATGAAGGTGTCGCCGAT	*SpeI* site added, for fragment GA20
RtsF-Sa	TAGAGCTCGCAATGGTGAGAGTTGCTGCCG	*SacI* site added, for fragment of RTS
RtsR-Ga	CCATGAGATTGGAGATGAGAAATCAGAGCGAGGTGGAGCAGC	*OsGA20ox2*-adapter added, for RTS
RtsF-B	ATGGATCCGCAATGGTGAGAGTTGCTGCCG	*BamHI* site added, for sense RTS-GA20
GaR-K	TAGGTACCACCATGAAGGTGTCGCCGAT	*KpnI* site added, for sense RTS-GA20 and DGF
GaF-Eat	GGTGAGTTCGGAAAGGGCAATCTCATCTCCAATCTCATGG	*OsEAT1*-adapter added, for fragment GA20
EatF-Sa	TAGAGCTCTTTGGAGCAAGAGGTTCCCC	*SacI* site added, for fragment of EAT
EatR-Ga	CCATGAGATTGGAGATGAGATTGCCCTTTCCGAACTCACC	*OsGA20ox2*-adapter added, for fragment EAT
EatF-B	ATGGATCCTTTGGAGCAAGAGGTTCCCC	*BamHI* site added, for fragment EAT
EatR-B	ACCATCTGACCCTAACTGGAGAGCTGAATCAC	Adapter added, for mutation of the *KnpI* site
GaF-K	GTTAGGGTCAGATGGTACTCAATCTCATCTAAT	Adapter added, for mutation of the *KnpI* site
AJCF	CTTACAGGAGTAGCAGCGGT	for PCR analysis of transgenic plants
AJCR	CTCGGCCACCATGAGATTGG	for PCR analysis of transgenic plants
Ga20QF	TGTCGCTGACGATCATGGAA	for qPCR analysis of *OsGA20ox2* gene
Ga20QR	TCCGCGAAGAACTCCCTGTA	for qPCR analysis of *OsGA20ox2* gene
EatQF1	AAGAAGGCCAACTCTCTGCT	for qPCR analysis of *OsEAT1* gene
EatQR1	CGCCGAACCTTCTGATACCT	for qPCR analysis of *OsEAT1* gene
UbqF	GCTCCGTGGCGGTATCAT	for qPCR analysis of ubiquitin gene
UbqR	CGGCAGTTGACAGCCCTAG	for qPCR analysis of ubiquitin gene
Ga3QF	GACGACTACCTCCTCTTCTGTGACGTG	for qPCR analysis of *OsGA3ox2* gene
Ga3QR	GAAGCCCGAGTCCGTGTGCGCGATG	for qPCR analysis of *OsGA3ox2* gene
Cps1F	GAACGTTTACCCGGTCGATC	for qPCR analysis of *OsCPS1* gene
Cps1R	CTTCAGTCCAGTGCCTGTTG	for qPCR analysis of *OsCPS1* gene

Supplementary Table S4 Nucleotide sequence of the fragments in the RNAi vectors

Fragment	Nucleotide sequence
EAT in sense	GATCCAGCTATCTGCACTATACCAGATCATATCATCAACCATCAGTTTAGCGAAGATCCACAAAAC<br>ATATTGGTGGAGCAACAGATCCAGCAGTATGATTCTGCACTTTATCCAAATGGTGTTTACACACCT<br>GCACCAGATCTCCTTAATCTTATGCAGTGCACAATGGCTCCAGCATTCCCGGCAACGACATCCGTA<br>TTCGGTGACACAACACTGAATGGTACTAACTATTTGGATCTTAACGGTGAACTTACAGGAGTAGCA<br>GCGGTTCCAGACAGTGGGAGTGGGTTGATGTTTGCTAGTGATTCAGCTCTCCAGTTAGGGTCAGAT<br>GGTACTCAATCTCATCTAATAAAGGATATCTGCCACTCGTTGCCCCAAAATTATGGGTTGTTTCCC<br>AGTGAGGACGAACGAGATGTGATTATTGGTGTTGGAAGTGGAGATCTTTTTCAGGAGATAGATGA<br>CAGGCAGTTTGATAGTGTACTTGAATGCAGGAGAGGGAAGGGTGAGTTCGGAAAGGGCAA
EAT in antisense	TTTGGAGCAAGAGGTTCCCCCAGTAGAAACTGCAAACTGGGATCCAGCTATCTGCACTATACCAG<br>ATCATATCATCAACCATCAGTTTAGCGAAGATCCACAAAACATATTGGTGGAGCAACAGATCCAG<br>CAGTATGATTCTGCACTTTATCCAAATGGTGTTTACACACCTGCACCAGATCTCCTTAATCTTATGC<br>AGTGCACAATGGCTCCAGCATTCCCGGCAACGACATCCGTATTCGGTGACACAACACTGAATGGT<br>ACTAACTATTTGGATCTTAACGGTGAACTTACAGGAGTAGCAGCGGTTCCAGACAGTGGGAGTGG<br>GTTGATGTTTGCTAGTGATTCAGCTCTCCAGTTAGGGTACCATGGTACTCAATCTCATCTAATAAA<br>GGATATCTGCCACTCGTTGCCCCAAAATTATGGGTTGTTTCCCAGTGAGGACGAACGAGATGTGAT<br>TATTGGTGTTGGAAGTGGAGATCTTTTTCAGGAGATAGATGACAGGCAGTTTGATAGTGTACTTGA<br>ATGCAGGAGAGGGAAGGGTGAGTTCGGAAAGGGCAA
GA20 in sense or antisense	TCTCATCTCCAATCTCATGGTGGCCGAGCACCCCACGCCACCACAGCCGCACCAACCACCGCCCAT<br>GGACTCCACCGCCGGCTCTGGCATTGCCGCCCCGGCGGCGGCGGCGGTGTGCGACCTGAGGATGG<br>AGCCCAAGATCCCGGAGCCATTCGTGTGGCCGAACGGCGACGCGAGGCCGGCGTCGGCGGCGGA<br>GCTGGACATGCCCGTGGTCGACGTGGGCGTGCTCCGCGACGGCGACGCCGAGGGGCTGCGCCGCG<br>CCGCGGCGCAGGTGGCCGCCGCGTGCGCCACGCACGGGTTCTTCCAGGTGTCCGAGCACGGCGTC<br>GACGCCGCTCTGGCGCGCGCCGCGCTCGACGGCGCCAGCGACTTCTTCCGCCTCCCGCTCGCCGAG<br>AAGCGCCGCGCGCGCCGCGTCCCGGGCACCGTGTCCGGCTACACCAGCGCCCACGCCGACCGCTT<br>CGCCTCCAAGCTCCCATGGAAGGAGACCCTCTCCTTCGGCTTCCACGACCGCGCCGCCGCCCCCGT<br>CGTCGCCGACTACTTCTCCAGCACCCTCGGCCCCGACTTCGCGCCAATGGGGTAATTAAAACGATG<br>GTGGACGACATTGCATTTCAAATTCAAAACAAATTCAAAACACACCGACCGAGATTATGCTGAAT<br>TCAAACGCGTTTGTGCGCGCAGGAGGGTGTACCAGAAGTACTGCGAGGAGATGAAGGAGCTGTCG<br>CTGACGATCATGGAACTCCTGGAGCTGAGCCTGGGCGTGGAGCGAGGCTACTACAGGGAGTTCTT<br>CGCGGACAGCAGCTCAATCATGCGGTGCAACTACTACCCGCCATGCCCGGAGCCGGAGCGGACGC<br>TCGGCACGGGCCCGCACTGCGACCCCACCGCCCTCACCATCCTCCTCCAGGACGACGTCGGCGGCC<br>TCGAGGTCCTCGTCGACGGCGAATGGCGCCCCGTCAGCCCCGTCCCCGGCGCCATGGTCATCAACA<br>TCGGCGACACCTTCATGGT
RTS in sense or antisense	GCAATGGTGAGAGTTGCTGCCGCCGCGGCGGTGCTCGTGCTGGCGGCGGCGGCGGCGGCGGCGGC<br>GGCCATGGCCGCCGAGCCGCCCACCGATGACGGCGCGGTCCGGGTGGCGGCGGGGCTGACGAAGT<br>GCGTGTCCGGGTGCGGTAGCAAGGTGACCTCCTGCTTGCTCGGCTGCTACGGCGGCGGCGGCGGC<br>GCCGCCGCCGCCGCGACGGCGATGCCGTTCTGCGTCATCGGCTGCACCAGCGACGTCTTGTCCTGC<br>GCCACCGGCTGCTCCACCTCGCTCTGATT

The internal *Kpn*I restriction site GGTACC (blue, in antisense EAT) was mutated as GGTCAG (yellow, in sense EAT). The 40 nucleotides (green) in antisense EAT was cut off in the sense EAT due to the *BamH*I restriction site GGATCC (underlined) during the cloning processes (Figure 3D-F).
